# Intra-Rater, Inter-Rater and Test-Retest Reliability of an Instrumented Timed Up and Go (iTUG) Test in Patients with Parkinson’s Disease

**DOI:** 10.1371/journal.pone.0151881

**Published:** 2016-03-21

**Authors:** Rob C. van Lummel, Stefan Walgaard, Markus A. Hobert, Walter Maetzler, Jaap H. van Dieën, Francisca Galindo-Garre, Caroline B. Terwee

**Affiliations:** 1 McRoberts BV, The Hague, The Netherlands; 2 MOVE Research Institute Amsterdam, Department of Human Movement Sciences, Vrije Universiteit Amsterdam, Amsterdam, The Netherlands; 3 Center for Neurology and Hertie Institute for Clinical Brain Research, Department of Neurodegeneration, University of Tuebingen, Tuebingen, Germany; 4 DZNE, German Center for Neurodegenerative Diseases, Tuebingen, Germany; 5 Department of Epidemiology and Biostatistics and the EMGO Institute for Health and Care Research, VU University Medical Centre, Amsterdam, The Netherlands; University Of Cambridge, UNITED KINGDOM

## Abstract

**Background:**

The “Timed Up and Go” (TUG) is a widely used measure of physical functioning in older people and in neurological populations, including Parkinson’s Disease. When using an inertial sensor measurement system (instrumented TUG [iTUG]), the individual components of the iTUG and the trunk kinematics can be measured separately, which may provide relevant additional information.

**Objective:**

The aim of this study was to determine intra-rater, inter-rater and test-retest reliability of the iTUG in patients with Parkinson’s Disease.

**Methods:**

Twenty eight PD patients, aged 50 years or older, were included. For the iTUG the DynaPort Hybrid (McRoberts, The Hague, The Netherlands) was worn at the lower back. The device measured acceleration and angular velocity in three directions at a rate of 100 samples/s. Patients performed the iTUG five times on two consecutive days. Repeated measurements by the same rater on the same day were used to calculate intra-rater reliability. Repeated measurements by different raters on the same day were used to calculate intra-rater and inter-rater reliability. Repeated measurements by the same rater on different days were used to calculate test-retest reliability.

**Results:**

Nineteen ICC values (15%) were ≥ 0.9 which is considered as excellent reliability. Sixty four ICC values (49%) were ≥ 0.70 and < 0.90 which is considered as good reliability. Thirty one ICC values (24%) were ≥ 0.50 and < 0.70, indicating moderate reliability. Sixteen ICC values (12%) were ≥ 0.30 and < 0.50 indicating poor reliability. Two ICT values (2%) were < 0.30 indicating very poor reliability.

**Conclusions:**

In conclusion, in patients with Parkinson’s disease the intra-rater, inter-rater, and test-retest reliability of the individual components of the instrumented TUG (iTUG) was excellent to good for total duration and for turning durations, and good to low for the sub durations and for the kinematics of the SiSt and StSi. The results of this fully automated analysis of instrumented TUG movements demonstrate that several reliable TUG parameters can be identified that provide a basis for a more precise, quantitative use of the TUG test, in clinical practice.

## Introduction

The ‘Timed Up and Go’ test (TUG) is a widely used measure of physical functioning (balance and mobility) in older people and in neurological populations, including Parkinson’s Disease (PD) [1–3]] It is a simple test that can be performed almost everywhere. The subject rises from an arm chair (Sit-to-Stand), walks 3 meters, returns to the chair and sit down again (Stand-to-Sit). The score given is the time taken in seconds to complete the test [4,5].

When the subject wears an inertial sensor measurement system, the individual components of the TUG can be measured separately. For example, in early stages of PD information on the components of each task, such as gait, turns or postural transitions (e.g. angular velocity and angular displacement) could reveal specific mobility problems. This may provide relevant information on the quality of movements. This version of the TUG is called an instrumented TUG, abbreviated as iTUG.

A few studies have used the iTUG in patients with Parkinson’s Disease (PD). Weiss et al. [6,7] found that several specific iTUG features, for example the amplitude range and slope in the accelerometer signal in anterior-posterior direction during the Sit-To-Stand and Stand-To-Sit time intervals, were different between patients with PD and healthy controls. Zampieri et al. [8] found differences between untreated patients with PD and healthy controls in several iTUG movement parameters, such as arm swing, cadence, trunk rotations, and turning velocity. Buchman et al. [9] reported that sub-tasks of the TUG were related to Parkinsonian signs and Herman et al. [10] and Mirelman et al. [11] demonstrated in PD patients that particular cognitive domains were related to iTUG subtasks. These studies suggest that the iTUG may be useful for studying mobility in patients with PD, to detect and quantify subtle differences in mobility and function and is only available using instrumentation. Further research should investigate the potential of the iTUG to identify PD, to monitor the progression of PD over time, and to asses the response and benefits to different therapeutic interventions.

Essential for these applications of the iTUG are good measurement properties. A high reliability is required to enable the measurement of small differences between patients with PD and healthy controls or changes in iTUG parameters over time. Measurement error may occur due to differences in attachment of the belt containing the accelerometers, differences in instructions given by the rater, or differences in behavior of the subjects over time. Subjects are usually instructed to walk at their comfortable speed, but the actual speed can fluctuate.

Little research has been performed on the measurement properties of the iTUG. As far as we know, only one study on the reliability of the iTUG with inertial sensors in PD patients has been reported. Salarian et al. found moderate to good intra-rater reliability for different iTUG parameters, in a sample of 18 subjects, 9 patients with PD and 9 controls [12].

The aim of this study therefore was to determine intra-rater, inter-rater and test-retest reliability of the iTUG in PD patients. The hypothesis was that test-retest reliability would be lower because patients with patients with Parkinson show unpredictable fluctuations of the disease [13].

## Methods

### Setting

Measurements were conducted at the outpatient clinic and ward of the Department of Neurodegenerative Diseases of the Center for Neurology of the University of Tübingen, the Gertrudis Klinik, Biskirchen, and a Physical Therapist Practice in ‘s-Gravenzande, The Netherlands. In order to establish if the patients were able to communicate well with the investigator and to understand and comply with the requirements of the study, clinical examination and absence of diagnosis of dementia was used. All patients provided written informed consent. The study protocol was approved by the Ethics Committee of the Medical Faculty of the University of Tübingen.

### Patients

Twenty eight patients with a diagnosis of Parkinson according to UK Brain-Bank criteria [14] were recruited. Mean age was 67.1 years (SD ± 8.3) and 22 patients were male. Median Hoehn & Yahr score was 3 (range 2–4). Patients needed to be able to walk 10 meters independently without ambulatory aids or assistance. Patients were tested during their subjective ‘‘on” phase. using their regular medication regimen [15].

### Procedures

All patients performed the iTUG five times on two consecutive days. On day 1, the first rater (A or B) explained and demonstrated the procedure. Then he attached the belt with the sensor and started the measurement by giving the start signal and operating the Remote Control (described below). One test trial (O) was performed in order to familiarize the patient with the procedure. This trial was not used for analysis. Morris et al [3] also removed the results of the first trial because it was abnormally slow. Then a second and third trial (AA or BB) were performed. After that, the first rater removed the belt. The second rater reattached the belt and the patients again performed two trials. After 24 hours, the whole procedure was repeated. Two raters (EvH and MH) performed all tests (raters A and B). The patients were assigned randomly to the test leaders. All possible combinations are visualized in Table 1.

**Table 1:** Order of measurements: O is a test trial, A is rater 1 and B is rater 2.

Day 1	Day 2
OAABB	OAABB
OAABB	OBBAA
OBBAA	OAABB
OBBAA	OBBAA

### Measurements

Participant’s trunk movements were measured with a small and light (87×45×14 mm, 74 grams) inertial sensor measurement system (DynaPort Hybrid, McRoberts, The Hague, The Netherlands), which was inserted in an elastic belt and positioned on the lower back near the spine. The device measured acceleration and angular velocity in three directions at a rate of 100 samples/s. Several Sit-to-Stand (STS) parameters can be identified that provide a basis for a more precise, quantitative study of STS performance in clinical practice [16,17]. The patients started the TUG while sitting on a regular, stable chair, with a height of 43–46 cm, without armrests. Patients were instructed to sit with their back against the back of the chair, feet placed on taped markers on the floor directly in front of the chair, with a distance of 43 cm between the feet and arms resting in their lap. Patients were instructed to rise from the chair (without using their arms) after the rater gave the starting signal, comfortably walk the clearly marked distance of 3 meter, turn around a cone, walk back to the chair and sit down with their back against the chair. The 3 meter walking distance was measured from the front of the chair to the middle of the cone. Markers in the signals of the inertial sensors were set at the start and the end of every trial using a remote control (McRoberts B.V.) which uses Bluetooth to connect with the DynaPort sensor. The rater also used a stopwatch to measure the time needed to perform the TUG, from the starting signal until the subject sat down on the chair again with the back against the back of the chair.

### Signal analysis

The iTUG was analyzed using commercially available software (DynaPort MoveTest, The Hague, The Netherlands). The total iTUG time was determined, as well as the following separate time intervals: sit to stand duration, walking first 3 meter duration, turning around the cone duration, walking second 3 meter duration, and turning before sitting duration and stand to sit duration. From the sit to stand and the stand to sit the separate flexion and extension durations were calculated. The maximum angular velocity during turning around the cone was calculated.

Start and end temporal events of the sit to walk and walk to sit phases were determined using peak detection of a low-pass filtered vertical acceleration signal. Maximal flexion angles of the sit to walk and walk to sit were determined using the trunk angle signal [18]. End and start temporal events of the sit to walk and walk to sit phases were determined as the first peak of the vertical acceleration signal after and before the maximum flexion angles and above the mean of the vertical acceleration signal. Global turning phases were determined using the low-pass filtered and squared angular velocity around the vertical axis. Start and end temporal events of the turning phases were determined using threshold detection based on low-pass filtering, squaring and differentiation of the angular velocity around the vertical axis.

**Fig 1:**
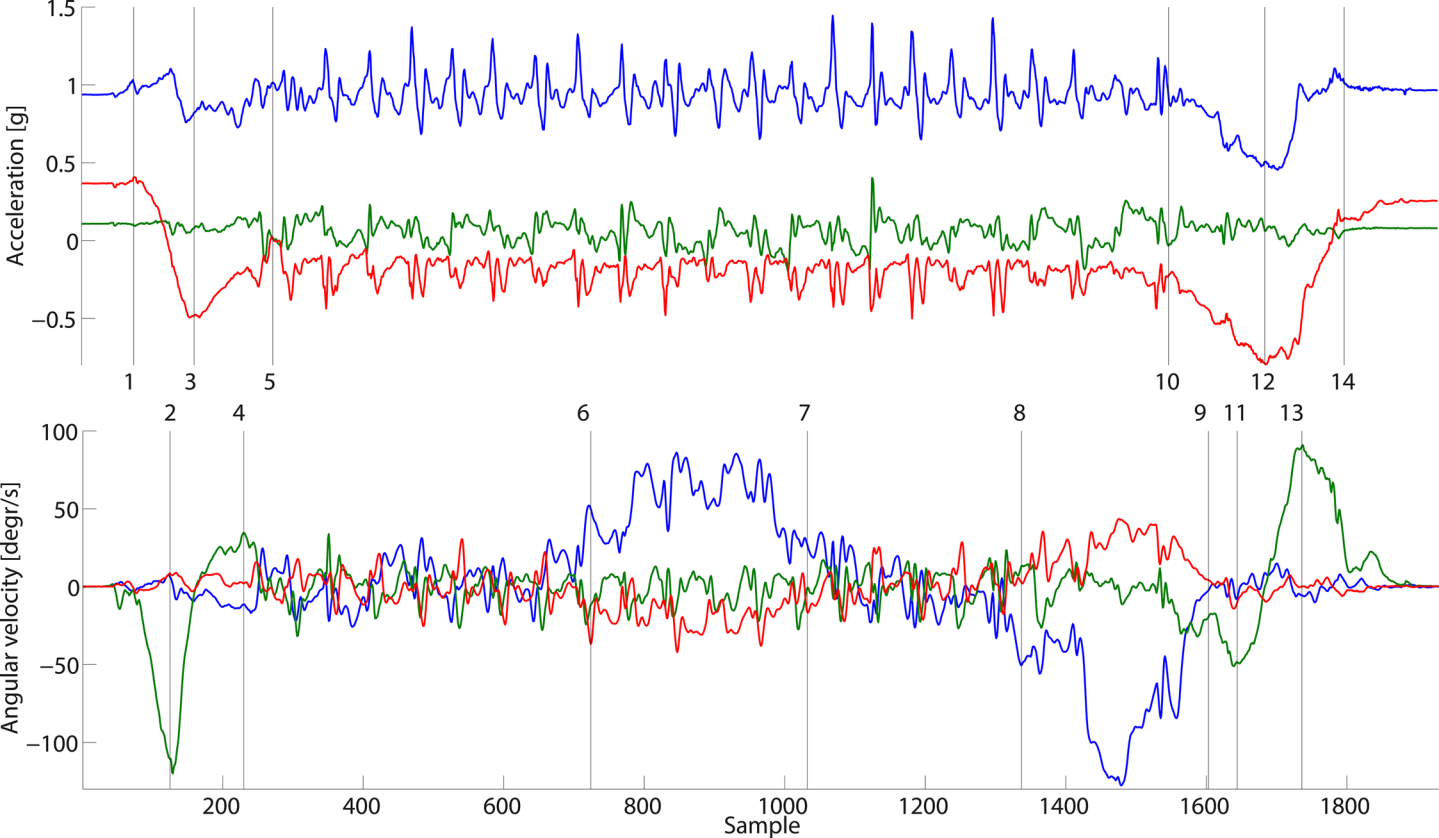
Raw data of the accelerometers on top and the gyroscopes at the bottom. 100 samples is one second. Blue are the vertical axes. green are the medio-lateral axes and red are the anterior-posterior axes. The numbers at the vertical lines correspond with the following events: 1 = Start SiSt. 2 = Maximum trunk flexion velocity SiSt. 3 = Maximum trunk flexion angle SiSt. 4 = Maximum trunk extension velocity SiSt. 5 = End SiSt. 6 = Start turn 1. 7 = End turn 1. 8 = Start turn 2. 9 = End turn 2. 10 = Start StSi. 11 = Maximum trunk flexion velocity StSi. 12 = Maximum trunk flexion angle StSi. 13 = Maximum trunk extension velocity StSi. 14 = End StSi.

From the trunk kinematics maximum angular velocity and angular displacement of the flexion and extension phase were calculated during the sit to stand movement and the stand to sit movement (Fig 1).

### Statistical analyses

Statistical differences between stopwatch and iTUG timing during Day 1 and Day 2 were tested using the dependent 2-group Wilcoxon Signed Rank Test, because most parameters were not normally distributed.

Measurement error was expressed in the Standard Error of Measurement (SEM) and the Smallest Detectable Change (SDC). The SEM value was derived from the error variance in the ICC formula. The SDC was calculated as 1.96*√2*SEM, which can be interpreted similar as the limits of agreement of the Bland and Altman method [19]. The Standard Error of Measurement (SEM) and the Smallest Detectable Change (SDC) of all variables (durations and kinematics) were presented in the same unit of measurement as the variable itself, for straightforward interpretation.

A single measures, two-way mixed model, type absolute intra-class correlation coefficient was used to calculate ICCs [20,21]. Intra-, inter-rater and test-retest reliability are expressed in Intra-class Correlation Coefficients (ICCs). The following equations for the ICC were applied. Each term refers to a variance component: p = patient, o = observer and m = moment.

Intra-rater reliability:

$$\frac{\sigma_{p}^{2}+\sigma_{po}^{2}+\sigma_{pd}^{2}}{\sigma_{p}^{2}+\sigma_{po}^{2}+\sigma_{pm}^{2}+\sigma_{pd}^{2}+\sigma_{residual}^{2}}$$

Inter-rater reliability:

$$\frac{\sigma_{p}^{2}+\sigma_{pm}^{2}+\sigma_{pd}^{2}}{\sigma_{p}^{2}+\sigma_{po}^{2}+\sigma_{pm}^{2}+\sigma_{pd}^{2}+\sigma_{residual}^{2}}$$

Test-retest reliability:

$$\frac{\sigma_{p}^{2}+\sigma_{po}^{2}+\sigma_{pm}^{2}}{\sigma_{p}^{2}+\sigma_{po}^{2}+\sigma_{pm}^{2}+\sigma_{pd}^{2}+\sigma_{residual}^{2}}$$

The familiarization trials (O) were not analyzed. Repeated measurements by the same rater on the same day (AA or BB) were used to calculate intra-rater reliability. Repeated measurements by different raters on the same day (AB or BA) were used to calculate inter-rater reliability. Repeated measurements by the same rater on different days (A-A or B-B) were used to calculate test-retest reliability.

We used thresholds, instead of significance, to asses reliability because they were less depending from the sample size. ICC’s were rounded at two decimals. An ICC of ≥ 0.90 was considered as excellent reliability, an ICC of ≥ 0.70 - < 0.90 was considered as good reliability, an ICC of ≥ 0.50 - < 0.70 was considered as moderate reliability, an ICC of ≥ 0.30 - < 0.50 was considered as poor reliability, an ICC of > 0.30 was considered as very poor reliability.

Data were analyzed using SPSS 20 for Windows (SPSS Inc., Chicago, USA).

## Results

Stopwatch timing was different from the iTUG timing for both raters on Day 1 and Day 2 (p<0.001) and between ICC’s calculated for Day 1 and Day 2 for the stopwatch and iTUG timing (p<0.001). The results for descriptive statistics of the durations, the SEM and the SDC are shown in Table 2.

**Table 2:** Mean, Standard deviation, Minimum, Maximum, Standard Error of Measurement and Smallest Detectable Change of the total time of the stopwatch, total time of the iTUG and individual components of the iTUG in seconds.

	Mean (s)	SD (s)	Min (s)	Max (s)	SEM (s)	SDC (s)
**Total durations**						
Stopwatch	11.80	3.11	7.80	18.54	0.61	1.69
iTUG	11.38	2.97	6.89	17.63	0.56	1.55
**Sit-to-Stand**						
SiSt Total	1.73	0.51	0.89	3.13	0.10	0.27
SiSt Flex	0.91	0.32	0.48	1.84	0.06	0.17
SiSt Ext	0.83	0.31	0.30	1.71	0.06	0.16
**Walks and Turns**						
Walk 1	2.19	0.82	0.95	4.24	0.16	0.43
Turn 1	2.65	0.57	1.84	4.39	0.11	0.30
Walk 2	1.81	0.66	0.76	3.18	0.13	0.35
Turn 2	2.33	0.48	1.71	3.82	0.09	0.25
**Stand-to-Sit**						
StSi Total	2.01	0.56	0.73	3.24	0.11	0.30
StSi Flex	1.03	0.39	0.24	1.71	0.07	0.20
StSi Ext	0.98	0.28	0.48	1.73	0.05	0.14

The results of descriptive statistics of the angular range (*θ*_flex_), the maximum angular velocity (ω_max_), the standard error of measurement (SEM), and the Smallest Detectible Change (SDC) are shown in Table 3.

**Table 3:** Descriptive values, SEM and SDC of the angular range (*θ*) in degrees (*°*) and the maximum angular velocity (ω_max_) in degrees per second (*°/s*) of the individual components of the TUG.

		Mean	SD	Min	Max	SEM	SDC
**Sit-to-Stand**							
SiSt Flex	*θ*_flex_ (*°*)	41.26	9.91	27.39	61.35	1.87	5.19
SiSt Flex	ω_max_ (*°/s*)	82.08	21.75	51.40	122.06	4.11	11.39
SiSt Ext	*θ*_flex_ (*°*)	21.20	7.45	5.86	41.39	1.41	3.90
SiSt Ext	ω_max_ (*°/s*)	32.63	10.00	17.77	58.03	1.89	5.24
**Turns**							
Turn 1	ω_max_ (*°/s*)	136.60	40.94	74.85	224.25	7.74	21.45
Turn 2	ω_max_ (*°/s*)	142.27	38.59	82.27	226.89	7.29	20.21
**Stand-to-Sit**							
StSi Flex	*θ*_flex_ (*°*)	18.90	8.00	4.13	32.12	1.51	4.19
StSi Flex	ω_max_ (*°/s*)	33.29	11.47	14.90	53.84	2.17	6.01
StSi Ext	*θ*_flex_ (*°*)	41.02	6.53	32.17	55.57	1.23	3.42
StSi Ext	ω_max_ (*°/s*)	77.32	14.42	56.82	110.62	2.73	7.55

The results of the intra-rater, inter-rater and test-retest reliability are shown in Table 4. Total duration, as measured with a stopwatch and as calculated from the kinematics were both highly reliable.

**Table 4:** Intra-rater, inter-rater and test-retest reliability of the TUG durations (s) and the trunk kinematics expressed in angular displacement of the flexion (*θ*_flex_) and the extension (*θ*_ext_) phase and the maximum angular velocity (ω_max_) of the TUG (n = 28).

**Durations**Intra-rater reliability (ICC) of iTUG durations and stopwatch duration											Total duration	
** **	SitSt	Flex	Ext	Walk 1	Turn 1	Walk 2	Turn 2	StSit	Flex	Ext	iTUG	TUG
Day 1	0.57	0.61	0.56	0.80	0.89	0.88	0.80	0.75	0.77	0.85	0.95	0.96
Day 2	0.62	0.37	0.57	0.88	0.89	0.86	0.89	0.81	0.78	0.79	0.98	0.97
Inter-rater reliability (ICC) of iTUG durations and stopwatch duration											Total duration	
** **	SitSt	Flex	Ext	Walk 1	Turn 1	Walk 2	Turn 2	StSit	Flex	Ext	iTUG	TUG
Day 1	0.52	0.58	0.56	0.84	0.90	0.91	0.80	0.74	0.73	0.77	0.95	0.96
Day 2	0.61	0.27	0.57	0.90	0.91	0.86	0.89	0.74	0.73	0.85	0.96	0.95
Test-retest reliability (ICC) of iTUG durations and stopwatch duration											Total duration	
** **	SitSt	Flex	Ext	Walk 1	Turn 1	Walk 2	Turn 2	StSit	Flex	Ext	iTUG	TUG
Day 1	0.47	0.59	0.42	0.86	0.84	0.71	0.71	0.75	0.67	0.73	0.88	0.90
Day 2	0.50	0.60	0.36	0.80	0.88	0.83	0.76	0.62	0.53	0.58	0.89	0.90
**Kinematics**												
Intra-rater reliability (ICC) of iTUG trunk kinematics												
** **	Sit to Stand Flex		Sit to Stand Ext		Turn 1	Turn 2	Stand to Sit Flex		Stand to Sit Ext			
	*θ*_flex_	ω_max_	*θ*_flex_	ω_max_	ω_max_	ω_max_	*θ*_flex_	ω_max_	*θ*_flex_	ω_max_		
Day 1	0.86	0.83	0.85	0.74	0.89	0.77	0.80	0.77	0.60	0.83		
Day 2	0.91	0.80	0.83	0.32	0.92	0.87	0.79	0.52	0.74	0.76		
Inter-rater reliability (ICC) of iTUG trunk kinematics												
** **	Sit to Stand Flex		Sit to Stand Ext		Turn 1	Turn 2	Stand to Sit Flex		Stand to Sit Ext			
	*θ*_flex_	ω_max_	*θ*_flex_	ω_max_	ω_max_	ω_max_	*θ*_flex_	ω_max_	*θ*_flex_	ω_max_		
Day 1	0.83	0.56	0.85	0.83	0.90	0.78	0.82	0.66	0.70	0.59		
Day 2	0.90	0.79	0.74	0.49	0.91	0.88	0.73	0.60	0.62	0.72		
Test-retest reliability (ICC) of iTUG trunk kinematics												
** **	Sit to Stand Flex		Sit to Stand Ext		Turn 1	Turn 2	Stand to Sit Flex		Stand to Sit Ext			
	*θ*_flex_	ω_max_	*θ*_flex_	ω_max_	ω_max_	ω_max_	*θ*_flex_	ω_max_	*θ*_flex_	ω_max_		
Day 1	0.59	0.66	0.47	0.38	0.84	0.83	0.63	0.52	0.45	0.47		
Day 2	0.60	0.52	0.47	0.39	0.88	0.73	0.53	0.41	0.33	0.18		

Nineteen ICC values (15%) were ≥ 0.9 which is considered as excellent reliability. Sixty four ICC values (49%) were ≥ 0.70 and < 0.90 which is considered as good reliability. Thirty one ICC values (24%) were ≥ 0.50 and < 0.70, indicating moderate reliability. Sixteen ICC values (12%) were ≥ 0.30 and < 0.50 indicating poor reliability. Two ICT values (2%) were < 0.30 indicating very poor reliability. The results clearly show that the reliability of total duration (range 0.88–0.95) and walk 1 and 2 (range 0.71–0.90) and turn 1 and 2 (range 0.71–0.91) is better than the reliability of the other parameters. Furthermore, the intra-rater and the inter-rater reliability were equal but the test-retest reliability was a bit lower.

## Discussion

In this study, intra-rater, inter-rater, and test-retest reliability were assessed in 28 patients with Parkinson’s disease. The intra-rater, inter-rater and test-retest reliability for the total duration, the walking and turning parts were good to excellent. Moderate reliability was found for the SiSt and StSi durations. The intra-rater and inter- reliability of the trunk kinematics showed good to excellent reliability. The test-retest reliability of the trunk kinematics showed moderate reliability for the SiSt and StSi and good reliability for the turns. In general the test-retest reliability was a bit lower than intra-rater and inter-rater reliability.

The attachment of the sensors, the instruction of the raters and the automated analysis of the individual components seem to have a small effect on the reliability because differences between intra-rater and inter-rater reliability were very small for the durations as well as the kinematics. The small differences between the intra-rater and the inter-rater scores were also comparable for the shorter sub parts of the TUG. Estimates of movement characteristics may suffer from errors due to discrepancies in accelerometer location. Rispens et al. [22] has shown that the differences in vertical sensor locations (L2-L5) on gait characteristics are small but some gait characteristics are more sensitive for mediolateral differences. This suggests that the sensors have to be attached accurately on the spine.

The data show a slightly lower test/retest reliability of most duration and kinematic parameters compared to the intra/rater and the inter/rater reliability. This shows that the behaviour of the subjects during consecutive days has more influence on the reliability than the behaviour of the raters. This could be affected by fluctuations of the movement symptoms of patients with PD.

We found seven other studies on the reliability of the normal TUG (studies on modified versions were omitted) [3,5,23–27] of which only one study was performed in PD patients [3]. One additional study was found on the reliability of an iTUG in PD patients and healthy controls [12]. The results of these studies are summarized in Table 5. These studies generally also show high inter- and intra-rater reliability of total TUG time. Test-retest reliability was low (ICC = 0.56) in the large study of Rockwood et al. [25]. However, the test-retest interval in this study was very large (mean 112 days), the tests were administered under different circumstances, and by different raters. Thus, despite the large sample size, the quality of this study is considered to be poor. Morris et al. (3) found an inter-rater reliability of 0.87–0.99 for total TUG time in Parkinson patients, which is comparable to our study (inter-rater ICC = 0.88–0.98). In the study of Salarian et al. [12] a poor intra-rater reliability (ICC = 0.04) was found for sit to stand duration, and high intra-rater reliability was found for turns (ICC = 0.89) and turn to sit (ICC = 0.84). We found a moderate intra-rater reliability for sit to stand duration on day 1 (ICC = 0.57), as well as on day 2 (ICC = 0.62). We also found higher intra-rater reliabilities for the turning parameters (ICC = 0.80–0.92). An explanation for this finding is that the turning phase can be detected from the available signals much easier than the other phases of the test.

**Table 5:** Results from earlier reliability studies.

Ref	TUG type	subjects	n	Intra-rater reliability (different days)	Inter-rater / reliability (same day)	Test-retest reliability(different days and different raters)	Intra-rater LoA* (sec)	Inter-rater LoA (sec)	Mean ± SD or median (range) sec
[5]	TUG	Elderly with a variety of medical diagnoses	2022	ICC = 0.99	ICC = 0.99		± 10	± 10	(11–128)
[19]	TUG	Unilateral lower limb amputation	32	r = 0.93			1.6 ± 10.2	0.5 ± 9.2	24.5 (9–102)
[18]	TUG	Community-dwelling elderly	1115			ICC = 0.56			14.0 (4–165)
[17]	TUG	Community-dwelling elderly	30	ICC = 0.93–0.99		ICC = 0.93–0.99			13.3
[16]	TUG	Elderly with impaired mobility	28	ICC = 0.68					
[20]	Mean of 2 TUGs	Inpatients on an orthopaedic rehabilitation ward	24			ICC = 0.80			22–104
[3]	TUG	PD patients	12		“Off” phase ICC = 0.87–0.99 “On” phase ICC = 0.99 **				“Off” phase 15–21 (10–45) “On” phase 13–15 (9–25)
[8]	iTUG	Early PD patients and healthy controls	12+12	Sit to stand ICC = 0.04 Turn ICC = 0.89 Turn to sit ICC = 0.84 (same day)					10.8 ± 0.5

* LoA is Limits of Agreement

**six raters rated the same video. so there was no between-patient variation

In the Salarian study [12], the only study in which inertial sensors have been used, intra-rater reliability has been studied. The walking part was longer (7 meter) than in the original TUG. The number of patients with PD was very low (n = 9) and the duration of the disease of the patients short (H & Y score between 1 and 2.5). Moreover, because both patients (n = 9) and healthy controls (n = 9) were included, the variability among subjects was larger. This artificially increases the reliability and decreases the generalizability of the results to future applications of the test in patients with PD only [28].

The results of our study should be interpreted with caution because of the relatively small sample size. We intend to collect more data in future studies. In addition, we intend to analyse more parameters, such as gait parameters and postural transitions (e.g. cadence, and number of steps). This may provide relevant information about the quality of movement. For example, in early stages of PD information on the components of each task, such as gait or postural transitions, could reveal specific mobility problems. The total duration taken with a stopwatch was a bit longer and the SD, SEM and SDC were larger than for the total iTUG duration (Table 2). Little is known about the accuracy of manually recorded time during performance tests. More research comparing these differences is necessary. There might be a difference between the start signal of the test leader and the start of the movement because of different reaction times of the participants. The observed difference may also be related to the accuracy of the test leader, who has to mark the start and stop of the movement and supervise the participant simultaneously.

In conclusion, in patients with Parkinson’s disease the intra-rater, inter-rater, and test-retest reliability of the individual components of the instrumented TUG (iTUG) was excellent to good for total duration and for turning durations, and good to low for the sub durations and the kinematics of the SiSt and StSi. The results of this fully automated analysis of instrumented TUG movements demonstrate that several reliable TUG parameters can be identified that provide a basis for a more precise, quantitative use of the TUG test, in clinical practice.
